# Developing digital applications for tailored communication in orthopaedics using a Research through Design approach

**DOI:** 10.1177/2055207618824919

**Published:** 2019-01-24

**Authors:** Bob Groeneveld, Marijke Melles, Stephan Vehmeijer, Nina Mathijssen, Tessa Dekkers, Richard Goossens

**Affiliations:** 1 Delft University of Technology, The Netherlands; 2 Reinier de Graaf Hospital, The Netherlands

**Keywords:** Patient engagement, patient education, prototype evaluation, design knowledge

## Abstract

**Objective:**

Tailored communication and information provision is expected to contribute to patient-centred care (PCC) in total hip arthroplasty (THA). In previous research, three subgroups of THA patients were identified that are similar in their clinical, psychological and communication characteristics. Preliminary subgroup-specific design guidelines were also formulated. Using these insights as a starting point, a theoretical framework was developed for tailored information provision and communication using digital applications. This study aims to refine the framework as well as subgroup-specific design guidelines for digital applications.

**Methods:**

This study uses a Research through Design (RtD) approach, generating insights both from the development and evaluation of prototypes in the early design stage. Paper-based prototypes will be made for each subgroup and evaluated with patients and care providers. Semi-structured interviews are held with participants exploring their experiences with the prototype. A quasi-experiment with a non-random control cohort is used to validate the qualitative findings. Post-surgery consultations with and without prototype are videotaped and scored using a structured instrument.

**Results:**

A design diary will be used to summarize design decisions and considerations. Feedback from participants is analysed inductively. Adaptations in subgroup-specific guidelines will be based on comparison of verbal feedback and descriptive statistics from consultations with and without prototype.

**Conclusions:**

Although mixed-method feasibility studies of digital health interventions are common, this protocol also considers the utility of the early design process and the designer’s perspective for realizing PCC and tailored care.

## Introduction

The utility and value of patient-centred care (PCC) is widely recognized. In patient-centred care, the patient is seen as a person with his or her own needs and characteristics; patient-centred communication (or interaction) is realized when care providers actively seek and discuss the patient’s perspective.^1^ Research has shown that PCC contributes to patient satisfaction,^2,3^ positive health outcomes^2^ and efficiency of care.^2,4^ This makes PCC a key quality indicator of healthcare quality and PCC is thus of competitive advantage for healthcare providers.^2,3,5^

This study focuses on PCC in relation to patients receiving total hip arthroplasty (THA, or a hip replacement). For this patient group communication and information provision has been shown to be particularly important,^6,7^ because THA is an elective procedure and therefor a conscious and carefully planned choice. Patients with osteoarthritis opt for a hip replacement at some point in time, usually after deliberation with an orthopaedic surgeon, and the surgery as well as recovery period are well planned. So in order to manage patient expectations pre-surgery and expectation fulfilment post-surgery, communication in THA can and should be patient centred.^8^ However, differences between patients in a variety of factors can influence what a patient might perceive as ‘good’ communication or information provision. Refining the process of patient care and communication in a way that reflects these differences is central to further advancing PCC and improving the patient experience in THA.^3^

## Definition of THA patient subgroups

Although no two patients are identical, we can expect that there will be commonalities in terms of a patients’ characteristics, preferences and needs, in relation to the THA process. To investigate how we could utilize such commonalities – and subsequently group THA patients according to such factors – we distributed a survey among 191 patients who had recently undergone a total knee or hip replacement surgery. Hip and knee surgery patients are similar in their communication needs,^7^ and were pooled together to increase sample size. In the survey, we assessed patients’ clinical, psychological and communication characteristics using a series of validated questionnaires measuring quality of life,^7^ self-perceived health status,^9^ pain,^10^ anxiety,^11,12^ tendency to catastrophize pain,^13^ coping style,^14^ communication skills,^15^ communication preferences^16^ and self-efficacy for health information.^17^ We used the resulting data set to identify clusters of patients in a series of unsupervised and supervised machine learning methods, including cluster analysis^18,19^ and recursive partitioning.^20,21^

This resulted in the identification of three subgroups: Subgroup A (44% of the study population) consisted of individuals with poor preoperative clinical status, who reported a diverse set of coping styles (e.g. active coping, planning, seeking support in others, self-distraction) and strong preferences towards communication, particularly discussing personal circumstances; subgroup B (33%) had a good preoperative clinical status and quality of life, reported limited strategies for coping and found patient–provider communication of lesser importance, with the exception of a need for open information; subgroup C (24%) was significantly older and more anxious. They reported coping behaviour that was distinct from other patients (e.g. coping through religion) and were less skilled and self-efficacious in communication about health.

## Framework for tailored communication and information provision

**Figure 1.**
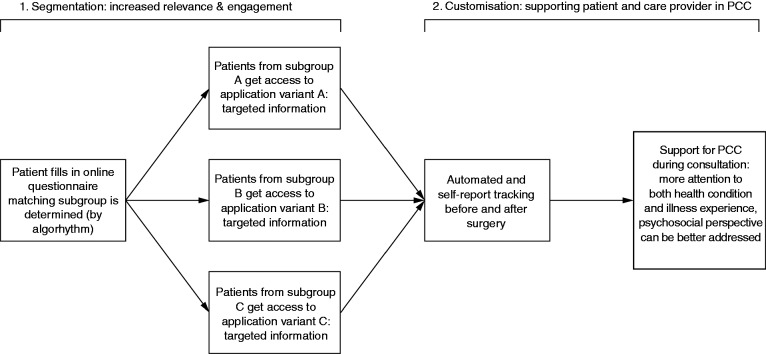
Framework for tailored communication and information provision in THA.

Based on the identified patient subgroups and earlier inquiries into the needs and experiences of THA patients,^22^ we developed a theoretical framework to be used as a blueprint for digital applications (such as a website or smartphone application) that support tailored communication and information provision for these patients. Figure 1 illustrates this framework. It consists of two steps: segmentation and customization. In Step 1 (segmentation), the patient completes a digital questionnaire. Based on the responses, the application determines which subgroup is the best match for the patient. The patient then receives access to a variant of the application designed for this specific subgroup. Adaptations in the application include the way that information is presented, labelled, or structured. We expect that this will increase the initial relevance of the application for the patient, and enhance engagement with the application as a result. In Step 2 (customization), the patient is offered self-tracking functions (such as textual or audio diaries, daily step count monitoring, or daily pain experience indication) to record their experienced recovery and specific questions that they may have for healthcare providers based on their experiences. This customized input is expected to enhance PCC through the interaction between patients and care providers. For instance, the care provider can give information and feedback during a consultation based on patient-specific data that the patient gathered in the week before that consultation.

Our framework is based on patient segmentation (the division of a generic target population into smaller subgroups), followed by customization (specific adaptations for individual members of each subgroup), in order to tailor to the needs of an individual patient. In this case, the THA patient population is segmented into three subgroups, and the application is subsequently further customized for each patient based on their input over time. Traditionally, distinction is made between communications that are targeted towards groups of people and those that are tailored towards specific individuals. However, Hawkins et al.^23^ argue that the concepts of segmentation and customization are more useful than this model of labelling communications either as ‘targeted’ or ‘tailored’ because a clear distinction between these levels of adaptation is problematic. In our framework, segmentation is applied to increase the initial relevance of the content, which is intended to facilitate engagement of patients with the application.^23,24^ Next, by using the self-tracking functions of the application, patients can reflect on their recovery process and customize the content of the application. During consultations, this information can promote the patient’s perspective on the recovery, which is seen as one of the pillars of PCC.^25^ Healthcare providers can use this information to give individualized feedback or specific information; functions which can be considered as tailoring strategies.^23^

## Research approach

In order to refine the framework described above (Figure 1) as well as subgroup-specific design guidelines for digital applications, this study uses a Research through Design (RtD) approach. RtD is an appropriate research approach to study the features, acceptance, and impact (three factors that are highly interdependent) of a design (in our case, a digital application). RtD is defined as knowledge generation through development as well as user evaluation of prototypes.^26^ In addition, the research process is an iterative one, and evaluation of a first prototype allows new insights in order to subsequently modify and improve the design.^27^ In our study, the prototype development process itself will lead to new insights, questions and issues surrounding the use of patient subgroups in the design of tailored healthcare communication.^26^ Other social or ethical issues surrounding the development of digital applications may also arise, such as a negative association with patients being divided into subgroups or issues surrounding data ownership and sharing.^28^ Through studying how these are addressed in the design process, such issues may be better anticipated in future projects.

**Figure 2.**
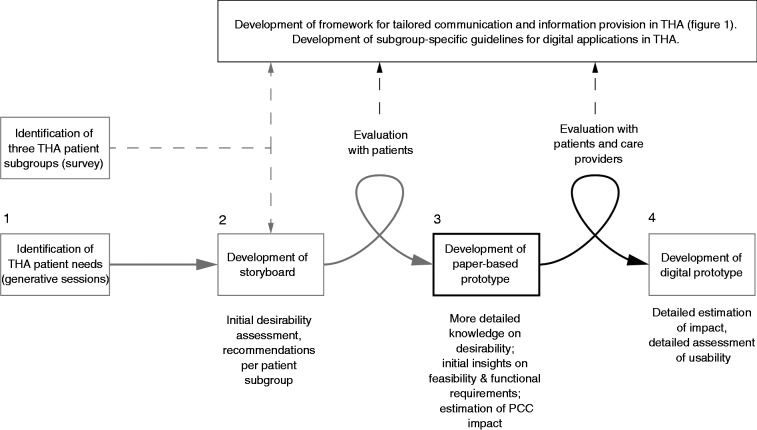
Development process of a digital application for tailored communication in THA. From left to right: (1) generative sessions with patients (in which patients share experiences from the past and hopes for the future through designerly activities^31^); (2) a storyboard for the digital application with evaluations of this storyboard by patients, taking into account the three THA patient subgroups; (3) development of the paper-based prototype and evaluation by patients and care providers; (4) development of the digital prototype. Knowledge goals are described under each step. Steps 2, 3, and 4 lead to insights for the development of both the framework described in Figure 1 as well as subgroup-specific guidelines for digital applications. Step 3 forms the subject of this protocol.

In this RtD project, User-Centred Design (UCD) principles are applied to create and evaluate prototypes. End-user needs and characteristics are considered from the start of product development, and users are actively involved throughout the design process.^29,30^ The current framework is also iteratively developed and based on several rounds of preliminary designs and evaluations from target users (Figure 2, steps 1 and 2).

In the study presented here (Figure 2, step 3), we make use of paper-based prototypes. Paper-based prototypes are often used in the early stages of digital user interface design, before the implementation of software starts.^32^ The architecture and functionalities of a specific application are mostly undetermined at this stage, and paper-based prototyping allows developers to quickly define, test and refine a design. In our study, information and self-tracking options for each subgroup can be quickly tested and easily adapted, and this way a paper-based prototype is an efficient way of testing whether – and if so, under what conditions – the desired effects of segmentation and customization described in Figure 1 could be realized. Moreover, research has demonstrated that, usually, target users (in our case patients and care providers) provide the same amount and type of feedback to a paper-based prototype as compared to a digital prototype.^33,34^

This study will provide insight into which design features are necessary and appropriate, serving as a basis for developing a digital prototype (Figure 2, step 4). Usability of the digital application within specific criteria for human–computer interaction (such as discoverability of functions, flow and structure of a digital application) will be evaluated at a later stage.

## Study objectives

This study aims to refine (a) the framework for tailored communication and information provision in THA by digital applications (Figure 1) and (b) the subgroup-specific design guidelines for digital applications in THA. To reach this goal, we will create and evaluate paper-based prototypes of a digital application for tailored information provision and communication in THA, based on segmentation and customization strategies. Specifically, we will define and implement several subgroup-specific features in the prototypes and evaluate the acceptance of the prototypes as well as their impact on PCC during post-surgery consultations.

## Methods

### Study design

**Figure 3.**
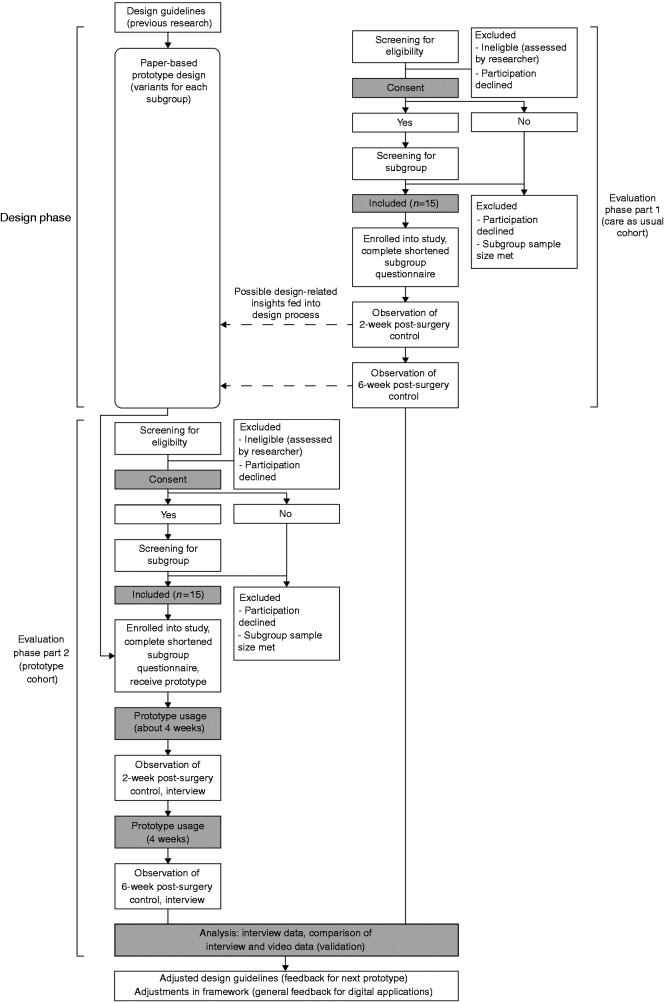
Study flow diagram. In the design phase, paper-based prototypes are designed for each subgroup. In the evaluation phase, 15 THA patients and four healthcare providers will use and evaluate the prototypes before and after surgery. A quasi-experiment with a non-random control cohort is performed to validate (through triangulation) the impact of using the prototype on care provider behaviour. The control cohort runs parallel to the design phase (see ‘Evaluation phase’ for the rationale for this).

This study uses an RtD approach consisting of two phases: a design phase and an evaluation phase. Figure 3 shows our study flow diagram illustrating these phases and the different activities within each phase. In the design phase, three paper-based prototype variants will be created that match characteristics and preferences of each THA subgroup, following the framework in Figure 1. The prototypes will consist of several features related to THA information provision and can be used throughout an extended period (i.e. several weeks). In the evaluation phase, 15 THA patients and 4 healthcare providers will use and evaluate the prototypes after surgery. A partially mixed concurrent design is adopted^35^ (see Figure 3): Semi-structured interviews with participants constitute the primary source of data collection. The interviews will be conducted following the consultations in weeks 2 and 6 after surgery, and they will explore the user experience and perceived impact of the prototypes on the communication with healthcare providers.^36^ This perceived impact is validated through triangulation^37^ in a quasi-experiment with a non-random control cohort: post-surgery consultations are video recorded in prototype users and a control group, and these observations are quantitatively compared in order to estimate the observed impact of using a prototype on PCC. This observed impact is contrasted to perceptions by patients and healthcare providers.

### Ethical review

A Dutch version of this research protocol was examined by the Medical Ethical Examination Committee of the province of South Holland, the Netherlands (file 17 – 146). It was decided by the committee (3 January 2018) that the Dutch law concerning research involving human beings (Dutch abbreviation *WMO*) does not apply to this protocol, and the need for formal approval was waived.

### Study setting

The study will be carried out at the Department of Orthopaedics of the Reinier de Graaf Hospital in Delft, the Netherlands (481 beds). This hospital is part of a more extensive network in the province of South Holland, providing services to around 450,000 people in the region. This non-academic training hospital has a strong focus on research and teaching activities. The department primarily serves THA patients that live in the region, but also regularly receives patients from other parts of the country that opt to have the procedure done in Delft.

### Design phase (paper-based prototype development)

In the first phase of this study, paper-based prototypes will be developed. Three variants will be created that match the characteristics and preferences of the three respective THA patient subgroups. This phase is discussed in more detail below.

### Main features of paper-based prototypes

**Figure 4.**
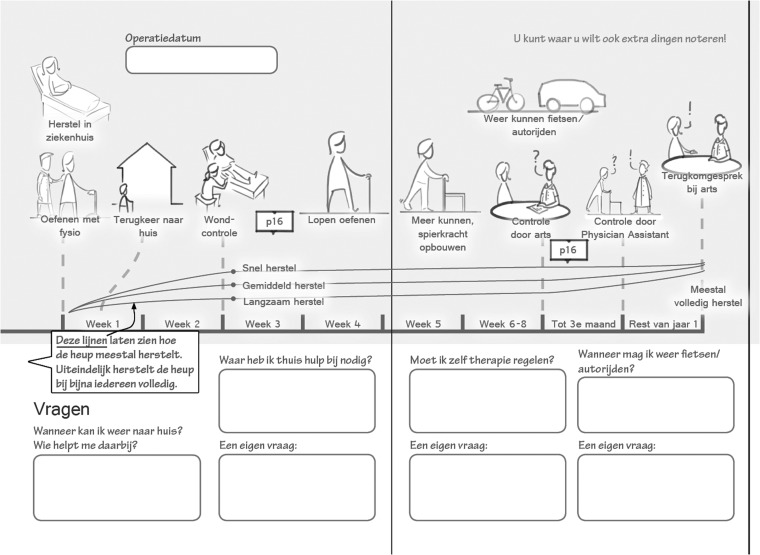
Impression of timeline in the paper-based prototype (Table 1, feature 1). The aim of this timeline is to support patient-care provider communication regarding patient expectations in preparatory consultations before surgery. In the top half, the timeline visualizes the process of recovery up to one year after surgery. In the lower part there is room to fill in predefined questions.

**Figure 5.**
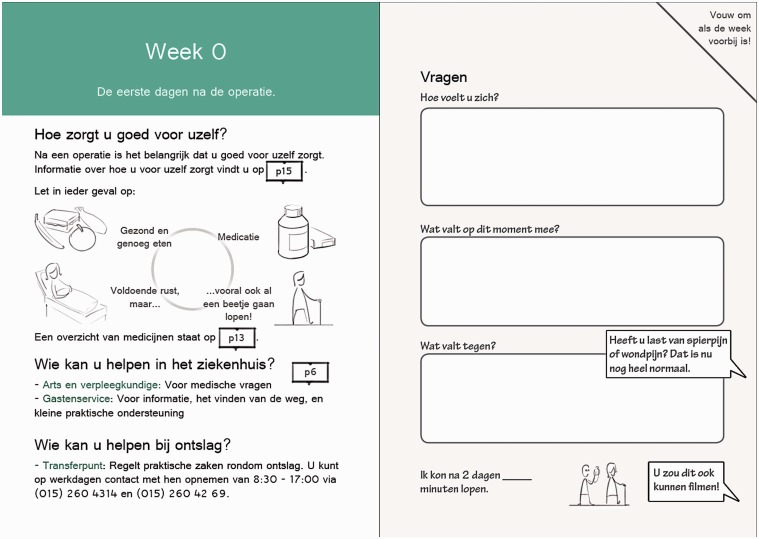
Impression of weekly information (left) and log book questions (right) form the second part of the paper-based prototype (Table 1, features 2 and 3 respectively). During the first six weeks after surgery, the prototype may contain information and questions for each week. The logbook aims to facilitate self-monitoring and active feedback seeking in patients, allowing them to track their progress.

Previous inquiries into the needs of patients (Figure 2, steps 1 and 2)^31^ led to a rich array of design-related insights, which resulted in the starting points and main features of each paper-based prototype: (a) a timeline providing an overview of the rehabilitation process after surgery (impression in Figure 4); (b) weekly information for the first six weeks after surgery; and (c) weekly questions and fill-in fields for the first six weeks after surgery (impression in Figure 5). Table 1 details the features of the prototypes, including an explanation and the intended effects of each feature. The content of the prototypes will be based on a generic patient information handout used at the study setting. A detailed account of how the previous design phases informed and inspired these features and starting points can be found elsewhere.^38^

**Table 1.** Main features of prototypes, explanation and intended effects. Variants of each feature are implemented in each prototype, to match preferences and characteristics of each subgroup.^38^

Prototype feature	Explanation of feature	Intended effects
1. Overview timeline depicting the rehabilitation process after surgery.	Patient and care provider can discuss the timeline of rehabilitation and patient expectations beforehand.	Manage patient expectations through feedback; answer specific questions.
2. Weekly information based on frequently occurring problems and questions (first six weeks after surgery).	Each week, the prototype offers relevant information concerning rehabilitation and recovery.	Emphasize that rehabilitation takes time; provide relevant information at the appropriate time.
3. Weekly questions and prompts (first six weeks after surgery).	Questions and prompts are provided for the patient to record and track their progress and experiences over time.	Facilitate self-monitoring and reflection in patients; illustrate patient recovery over time.

Each paper-based prototype will contain the features outlined in Table 1, but there will be differences among each prototype in how the features are implemented. A prototype for one subgroup may for instance contain a more informal framing of the weekly information (feature 2) and the fill-in fields (features 3) may be more structured compared to the prototypes for other subgroups.

### Procedure

Three variations of the paper-based prototype will be designed, with adaptations per subgroup. These adaptations are based on predefined characteristics identified from the survey study (see ‘Definition of patient subgroups’ in the Introduction), as well as patient feedback (*n* = 12) on a storyboard version of the design (Figure 2, step 2). The insights gathered in these steps were summarized into preliminary guidelines for adapting each prototype variant to the preferences of the corresponding subgroup. Guidelines are formulated for the design in general, as well as the timeline (Table 1, feature 1) and log book (Table 1, features 2 and 3). The specific guidelines are published elsewhere.^38^

### Outcomes

One outcome of the design phase will be three paper-based prototypes, corresponding to the needs and characteristics of the three THA patient subgroups, as well as an overview of considerations that underlie this design output. In addition, in order to formulate potential design opportunities, constraints and reflections based on the process of prototype development, a structured diary will be used. For the type of project described in this protocol, a structured diary is an acceptable option for detailed data collection.^39^ Data entries will be made following pretested guidelines, and entries will be made for each day that design activities are carried out, with links to design materials where relevant. Data will be prepared for analysis by numbering and labelling each entry in terms of a content analysis encoding scheme.^40^

Design diary entries and metadata (entry number and initial activity type code) will be logged in a spreadsheet using Microsoft Excel. The diary is reread and critical events are listed. Based on further analysis of entries, the predefined set of category codes is adapted where necessary.^40^ A general inductive approach is used to summarize and explain design activity code-by-code. Particular attention will be paid to suggested options and opportunities for design features, as well as pitfalls or criticisms of the prototype design.

### Evaluation phase

In the evaluation phase, the prototypes will be given to THA patients of each corresponding subgroup. Feedback by patients and care providers on the prototypes provided by means of semi-structured interviews will be used to explore the acceptance and estimated impact of the design in general, as well as specific design features. Video observations of post-surgery consultations will be analysed and compared to care as usual, in order to validate the estimated impact of the prototype on PCC.

### Sample

Two consecutive samples of patients will be recruited for the study, one for control observations and one for prototype use. Both cohorts will consist of 15 patients. Within each cohort, there will be five patients from each subgroup. We considered setting up a small-scale randomized trial with simultaneous recruitment and randomized allocation into either prototype or control group; however, we reasoned that additional design-related insights might emerge from the observations of care as usual, so the control cohort for care as usual will be recruited and observed first.

For the prototype cohort, five patients of each corresponding subgroup will test a corresponding prototype. As a rule of thumb, it is good practice in user evaluations to include at least five participants from each homogenous group in formative testing (i.e. testing with unfinished designs in order to improve the design).^32^ As we have defined three groups, the sample for one prototype evaluation should consist of at least 15 patients. In addition to patients, three to five care providers (one or two orthopaedic surgeons, one or two medical consultants, a physician assistant) will be included to observe interactions with patients.

### Recruitment

Eligible participants are elective THA patients who opted to undergo surgery at the study setting. For both cohorts, surgery should take place a maximum of two weeks before recruitment. Exclusion criteria for patients include insufficient comprehension of the Dutch or English language or insufficient mental capability to fill out a 10-minute questionnaire, as assessed by the researcher. Eligible healthcare providers are professionals involved in the THA patient journey in the post-surgery recovery period until week 6 after surgery. Because the design proposal and intended prototypes can be used by surgeons, nurses and physician assistants, these care providers are all eligible to participate in the study.

The first author has responsibility for the recruitment of participants. In consultation with hospital partners, the first author or selected healthcare providers (e.g. medical consultant or research nurse) will inform patients about the study and ask if they can be contacted for participation. Non-respondents will be called again after three days.

### Screening and assignment to subgroups

A screening instrument will be used to make an initial classification of respondents, and a shortened version of the survey described in the introduction (see ‘Definition of THA patient subgroups’) will then be used to make the final classification of patients into subgroups. Only patients that are included by the screening instrument fill in the shortened survey, which reduces patient burden. For instance, if sample size requirements are met for two of three subgroups and inclusion is only needed for one more subgroup, we can exclude individual patients based on the screening instrument if this instrument indicates that the patient does not seem to belong to the ‘incomplete’ subgroup.

The screening instrument and shortened survey were developed in a way such that they only included the variables that best distinguished between subgroups. In the screening instrument, these variables are measured using three questions, asking respondents to signify a presence/absence of (a) coping by planning (‘I've been trying to come up with a strategy about what to do’); (b) feeling helpless when in pain (‘When I’m in pain, I feel I can’t stand it anymore’); and (c) preference for completely open information provision (‘Your physician should always tell you everything about your illness, even if it is unpleasant’). The screening instrument was 76% accurate to classify patients into subgroups and performed slightly better in the classification of patients from subgroups A and B compared to subgroup C.

For the shortened survey, the subset of variables includes age, anxiety,^11,12^ pain catastrophizing,^13^ coping style,^14^ skill in active-disease related communication^15^ and preference for open communication.^16^ Eliminating non-discriminating variables reduced the survey length from 40 to 10 min.

In the case that a patient is allocated to a group which has already reached its sample size requirements, participation will be discontinued; the patient can still be kept informed about the study if they wish to be. The above process is continued until enough participants are allocated to each group.

### Procedure

The prototypes will be embedded in the THA care pathway at Reinier de Graaf Hospital in an as unobtrusive manner as possible. The prototypes will impose no restrictions to optimal or usual care. This also means that patients and care providers are free to use, or discontinue using, the prototype during consultations or at home. Participants are also free to use whichever features of the prototype they deem relevant. Participants are however requested to report discontinued or altered use to the researchers. Reasons for discontinued, incomplete or altered prototype use will be taken into account in iterating and improving the design.

To stimulate intervention adherence (i.e. the use of the prototype), a researcher will shortly explain the use of prototype to participants and will also be present in meetings where the prototype is used.

### Outcomes

Outcomes will include qualitative and quantitative insights regarding the use and evaluation of the prototypes. Interview data is gathered to obtain insights into both patient and care provider evaluation of booklet usability and perceived impact on the consultation.^41^ To validate the perceived impact, interactions between patients and healthcare providers are observed using a structured instrument to estimate the impact of the prototype on PCC. We expect the prototype to positively impact PCC, as it is likely that the patient and care provider will actively discuss the patient’s recovery experience when using the prototype before or during a consultation. Recognition of the patient perspective in such a way is considered one of the pillars of PCC.^25^

### Interviews with patients and care providers

After each consultation where a prototype is used (in weeks 2 and 6 after surgery, see also Figure 3), patients will be interviewed about their experiences with the prototype. Patients will be asked about their general experience and impressions at first (‘How did you experience using the prototype so far?’). Subsequently, specific questions will be asked regarding the different features described in Table 1 (‘What do you think are strong or weak aspects of this feature? What points for improvement can you think of for this feature?’ etc.). Follow-up questions will be asked based on answers given by participants (‘Can you elaborate on the answer you just gave regarding [general experience with prototype/a specific prototype function or feature]?’). Patients will also be asked to estimate the impact of using the prototype on their communication with the healthcare provider during the consultation (‘To what extent do you think the prototype did or did not influence the conversation in your post-surgery consultations? What makes you think this?’).

Healthcare providers will be asked to evaluate the use of the prototype and the overall interaction across all cases, and they will be asked to shortly explain this evaluation through similar questions as those described above.

### Video-based observations of consultations

For both the control and prototype cohorts, consultations in weeks 2 and 6 after surgery will be videotaped. These observations will be coded using the patient-centred behaviour coding instrument (PBCI).^1^ This instrument can be used to code physicians’ explorative communication behaviour in a consultation; specifically, it can be used to assess the behaviours that inhibit or facilitate patients to share their perspective on their health condition. There is a clear conceptual link between the behaviour that this instrument captures and the intended impact of the design and paper-based prototypes. In addition, the psychometric properties of this instrument seem to be favourable compared to other instruments.^42^

### Data management and analysis

Each participant will be assigned a study code to allow an anonymized comparison of results across subgroups and cohorts. Survey responses will be digitized in IBM SPSS^®^ version 22 for Windows; subgroup assignment is done with a custom script written in R for Windows. Observational and interview data will be processed using NVivo Pro 12 for Windows. Observational data will be collected with video recordings and researcher notes, and interviews will be audio recorded. Transcript excerpts will be double checked by the corresponding author and a second researcher for accuracy.

Interview data will be analysed inductively, in accordance with the guidelines of qualitative content analysis.^43^ Each transcript is segmented into ‘meaning units’, containing words, sentences or paragraphs that are related in terms of their content and context. To summarize the content, all meaning units are condensed and interpreted. These condensed meaning units are grouped into categories, which are then grouped into themes. Themes will be generated inductively, and may for instance concern prototype features, the interaction between the patient and the care provider, and patient or care provider experience of their interactions in general. Structures and themes will be identified for each subgroup of patients. The perceived impact on the consultation (from interview data) will be analysed separately as well.

To analyse the video observations, care provider behaviours will be analysed using the categories defined by the PCBI.^1^ Individual behaviour counts will be weighed based on categorical principal component analysis,^44^ and the weighted sum scores will represent overall care provider performance in terms of ‘facilitating’ or ‘inhibiting’ behaviour during the consultation. Consultation length will be controlled for by transforming the scores into behaviour rates per 10 min. Descriptive statistics and confidence intervals will be generated to estimate differences in facilitating and inhibiting behaviours for both post-surgery interactions.^45^ Quality of data coding will be promoted as follows: transcript excerpts or observational data will be coded by a second author for 10% of data. These analyses will be compared and discussed until agreement is reached (as much as possible). This will both be done to refine the observation coding (in a formative stage) and to assess interrater agreement.

Participants’ interview responses will then be validated using the quantitative comparisons of care provider behaviour. We will use triangulation to determine whether there is agreement, partial agreement, or disagreement between the qualitative and quantitative results.^37^ For example, patients may be very enthusiastic about the prototype and estimate that it positively impacts their communication with a care provider, but this impact may not be reflected in higher estimated PCC rates in videotaped consultations, compared to care as usual. Table 2 details various triangulation scenarios, and implications for adapted design guidelines.

**Table 2.** Meta-analysis and triangulation scenarios for study components in the evaluation phase.^37^

Qualitative results (interview data)	Quantitative results (video analysis data)	Possible conclusion	Possible implications for design guidelines
Patients/care providers are enthusiastic about the prototypes and/or feel that its use positively impacts communication	Clear difference between control and prototype groups in PCC (i.e. higher facilitating and/or lower inhibiting behaviour rates)	Agreement: Prototype performs as expected	Little or no adaptations to guidelines needed
Patients/care providers have many remarks on prototype, and/or do not feel that its use impacts communication during post-surgery consultations	Clear difference between control and prototype groups in PCC (i.e. higher facilitating and/or lower inhibiting behaviour rates)	Disagreement: Prototype performs as expected, but this is not perceived as such by users	Use same features in next prototype, but expand them or frame them differently
Patients/care providers are enthusiastic about the prototypes and/or feel that its use positively impacts communication	No (clear) difference between control and prototype groups in PCC (i.e. similar facilitating/inhibiting behaviour rates)	Disagreement: Prototype does not perform as expected, but users are satisfied with it	Expand features and functions in next prototype, in order to increase its impact
Patients/care providers have many remarks on prototype, and/or do not feel that its use impacts communication during post-surgery consultations	No (clear) difference between control and prototype groups in PCC (i.e. similar facilitating/inhibiting behaviour rates)	Agreement: Prototype does not perform as expected	Formulate new features or functions (perhaps even different objectives) for next prototype

## Discussion

This protocol uses an RtD approach in order to refine a framework and design guidelines for tailored information provision and communication applications in THA. Insights into the required features, acceptability and impact of the design are generated from both the development and evaluation of paper-based prototypes. Semi-structured interviews are held with participants concerning their experiences with the prototype and their estimated impact on post-surgery consultations, and a quasi-experiment with a non-random control cohort is used to validate the impact on PCC during consultations in weeks 2 and 6 after surgery.

To refine the framework (Figure 1) and subgroup-specific guidelines for the design of tailored digital applications, these combined outcomes will be critically reflected upon. This is common practice in design processes, where insights from prototype testing are used to improve a design.^27^ Special attention will be paid to criticisms from patients and care providers regarding ethical aspects or feasibility. In reflecting on the impact of the prototype on PCC, the comparison of perceived impact (qualitative interview data) and validation through video observations (quantitative video-observation data) will be used to make the final recommendations for future design iterations. Various triangulation scenarios and implications for adapted design guidelines are detailed in Table 2.

This protocol shows similarities with relatively common mixed-method protocols to study the feasibility and acceptability of digital interventions. Recent examples include a study using Facebook as a tool for people with serious mental illness,^46^ an application for women with pregestational diabetes,^47^ or the use of digital technologies by patients with musculoskeletal conditions in the waiting room.^48^ In addition to this type of study, our RtD approach considers the early stages of design and the perspective of the designer as valuable sources of knowledge. Reflections made in this early process by the designer, as well as users, can result in high-quality guidelines for creative practice. These types of insights are sometimes defined as ‘strong concepts’ or ‘intermediate-level knowledge’,^49^ i.e. specific types of interactions and design recommendations for specific target groups that can also be applied and evaluated in other (similar) contexts. A paper-based prototype is an efficient means to gather these insights at this early stage and can still result in valid user input for digital prototypes and the final application. Moreover, evaluating a digital prototype at this stage may confound the results as target users may, in general, prefer (or dislike) the concept of digital information provision.

This study also bears much similarity to the person-based approach for health behaviour change intervention development.^36^ This approach uses in-depth qualitative research in an iterative fashion throughout the development process, in order to make health behaviour change interventions more convincing and persuasive for users. Goal-based design guidelines are also set up from the early stages, to steer the development process. The approach goes beyond usability or feasibility testing, also looking at how users implement the behaviour change techniques. Similarly, in our Evaluation phase we examine the experience of users with the prototype in terms of both acceptability and impact on PCC. However, we also make use of a quantitative validation of the perceived impact, through video analysis of post-surgery consultations. In addition, even in early development stages we apply basic (paper-based) prototypes to evoke specific feedback and responses by end-users. Moreover, we let patients evaluate storyboard of design features in order to create the initial set of guidelines (Figure 2, step 2).^38^ This prototype-led research setup is not necessarily part of the person-based approach from the earliest stages. So while we agree that the person-based approach is a highly relevant and valuable addition to theory-based and evidence-based intervention development, the RtD protocol outlined in this protocol seems to add several elements to this approach.

This study has several limitations. Intensive observation and follow-up interviews with patients about the prototypes may introduce bias in behaviour during consultations and feedback on the prototypes. Also, sample sizes in this study are relatively small, which limits the generalizability of findings to the overall THA population and other contexts. Moreover, the use of a paper-based prototype for a digital application is useful in this design stage, but specific aspects such as navigation through the application should be tested with a digital prototype.

Nevertheless, we expect that this study will produce valuable and actionable insights for tailoring communication and information around THA using digital applications. As THA patients particularly value this aspect of care delivery, we expect that these applications will positively impact patient-centredness.
